# Porto-Sinusoidal Vascular Disease Associated to Oxaliplatin: An Entity to Think about It

**DOI:** 10.3390/cells8121506

**Published:** 2019-11-24

**Authors:** Angela Puente, Jose Ignacio Fortea, Carmen Del Pozo, Patricia Huelin, Maria Luisa Cagigal, Marina Serrano, Joaquin Cabezas, Maria Teresa Arias Loste, Paula Iruzubieta, Antonio Cuadrado, Susana Llerena, Carlos Lopez, Emilio Fábrega, Javier Crespo

**Affiliations:** 1 Digestive Disease Department, Marqués de Valdecilla University Hospital, Cantabria University, Av. Valdecilla s/n, 39008 Santander, Spain; joseignacio.fortea@scsalud.es (J.I.F.); carmendel.pozo@scsalud.es (C.D.P.); patricia.huelin@scsalud.es (P.H.); joaquin.cabezas@scsalud.es (J.C.); mteresa.arias@scsalud.es (M.T.A.L.); paula.iruzubieta@scsalud.es (P.I.); antonio.cuadrado@scsalud.es (A.C.); susana.llerena@scsalud.es (S.L.); emilio.fabrega@scsalud.es (E.F.); javier.crespo@scsalud.es (J.C.); 2 Health Research Institute Marques de Valdeciila, IDIVAL, 39011 Santander, Spain; 3 Pathology Department, Marqués de Valdecilla University Hospital, 39011 Santander, Spain; mluisa.cagigal@scsalud.es; 4 Oncology Department, Marques de Valdecilla, University Hospital, IDIVAL, 39011 Santander, Spain; marina.serrano@scsalud.es (M.S.); carlos.lopez@scsalud.es (C.L.)

**Keywords:** oxaliplatin, non-cirrhotic portal hypertension, porto sinusoidal vascular disease

## Abstract

Portal sinusoidal vascular disease is a presinusoidal cause of portal hypertension (PHT) of unknown etiology, characterized by typical manifestations of PHT (esophageal varices, ascites, portosystemic collaterals), plaquetopenia and splenomegaly with a gradient of portal pressure slightly increased, according to the presinusoidal nature of the PHT. A few cases in the literature have shown a relationship between oxaliplatin and the development of presinusoidal portal hypertension, years after the chemotherapy for colorectal cancer (therefore, different to sinusoidal obstruction syndrome). There are three mechanisms through which oxaliplatin can cause sinusoidal damage: (1) damage at the level of endothelial cells and stimulates the release of free radicals and depletion of glutathione transferase, with altering the integrity of the sinusoidal cells. The damage in the endothelial sinusoidal cells allows to erythrocytes to across into the Dissé space and formation of perisinusoidal fibrosis, (2) the appearance of nodular regenerative hyperplasia is favored by the chronic hypoxia of the centrilobular areas and, finally, (3) oxaliplatin can generate an obliteration of the blood capillaries and zones of parenchymal extinction. These three facts can develop, in a minority of cases, the appearance of a presinusoidal increase of portal pressure, which typically appears years after the completion of chemotherapy and sometimes is underdiagnosed until variceal bleeding, ascites or encephalopathy appear. The knowledge of this pathology is essential to be able to perform an early diagnostic and consult to the hepatologist.

## 1. Introduction

Idiopathic portal hypertension (IPH) is an uncommon cause of portal hypertension characterized by clinical portal hypertension in the absence of an identified cause such as cirrhosis. Typically, patients can develop varices, ascites, porto-systemic shunts, plaquetopenia and splenomegaly with a slight increase in portal pressure gradient (HVPG), non-advanced fibrosis in liver biopsy or liver stiffness measurement (LSM) and permeability of portal and suprahepatic vessels [1,2].

In the past, the terminology was changed from non-cirrhotic portal fibrosis to hepato-portal sclerosis, incomplete septal cirrhosis or regenerative nodular hyperplasia [3,4], without strong definition criteria. It has been suggested that IPH would be the top of an iceberg, in which a larger number of asymptomatic patients would have histologic lesions compatible with hepatoportal sclerosis, but only a small percentage would develop the symptomatic clinical picture. Recently, a group of experts on behalf of the VALDIG group (Vascular Liver Disease Interest Group) proposed new terminology, in order to find a name that reflects the description definition: porto-sinusoidal vascular disease [5] (PSVD).

The epidemiology of this entity is unknown; however, there is a predominance of males, with an average age of presentation at 40 years, [5]. The main etiological factors identified associated with the development of PSVD are immunological disorders, infections, human immunodeficiency virus and/or its treatments, drugs (azathioprine, oxaliplatin), toxins, genetic predisposition and thrombophilia [6,7,8,9]. In Western countries, a prevalence of underlying thrombophilic disorder has been reported in up to 40% of patients with PSVD [3]. It has also postulated a genetic basis for the disease. The presence of mutations in the gene DGUOK56 and in KCNN357 has recently been described in patients with PSVD; however, more studies are needed [10].

## 2. Porto-Sinusoidal Vascular Disease Associated to Oxaliplatin

### 2.1. Oxaliplatin and Liver Damage

Oxaliplatin, a third-generation derivative of platinum (platinum compounds belong to a family of platinum, Pt), is one of the main chemotherapeutic agents currently used as a neoadjuvant/adjuvant treatment in stage III and metastatic colon cancer, in combination with 5-fluorouracil (5-FU) (FOLFOX^®^) or capecitabine (XELOX^®^) [11]. It is also used in other cancers of the gastrointestinal tract such as gastric and pancreatic cancer [12,13].

Oxaliplatin is composed of a central Pt atom that forms a complex with 1,2-diaminocyclohexane (DACH) and an oxalate group. Although to a lesser extent than cisplatin (three time less), oxaliplatin is able to produce Pt-adducts in DNA strands (both drugs mainly create guanine-guanine intra strands). For this reason, oxaliplatin generates less neurotoxic than cisplatin; in fact, it has been observed that the amount of Pt-adduct is linear with the severity of peripheral neuropathy (few adducts generate less toxicity) [14]. Hematologic toxicities (neutropenia and thrombocytopenia) are also detected during oxaliplatin treatment.

Among the most frequent side effects associated with oxaliplatin is peripheral neuropathy, isolated alteration of liver function tests, splenomegaly and thrombocytopenia. The causal relationship with hepatic steatosis and sinusoidal obstruction syndrome (SOS) is relatively well known [15]. Rubbia-Brandt et al. showed for the first time, in 2004, in a cohort of 154 patients undergoing resection of liver metastases, an incidence of sinusoidal dilation and microscopic hemorrhage secondary to damage to the hepatic sinusoidal in 51% of the patients (all of them with neoadjuvant treatment with oxaliplatin) [16]. Several series of cases have confirmed the relationship between sinusoidal damage and treatment with oxaliplatin with incidence rates ranging between 19% and 52% [17,18]

**Figure 1:**
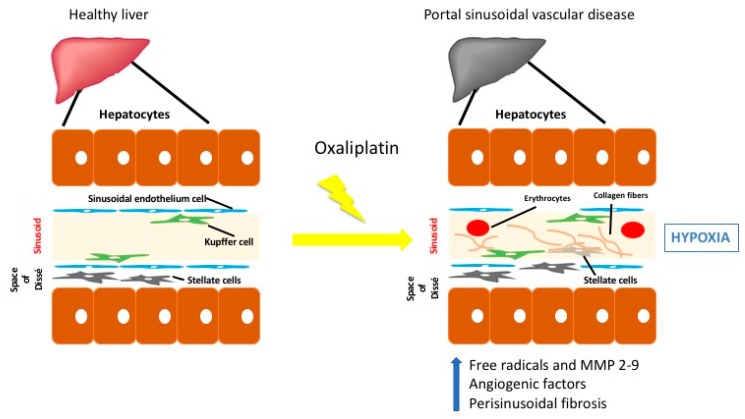
Proposed oxaliplatin liver injury mechanisms of sinusoidal damage: first, oxaliplatin increases porosity of the sinusoidal endothelium cellular fenestrations, stimulating the release of free radicals and depletion of glutathione transferase, followed by an increase of metalloproteinases (MMP-2 and MMP-9). This damage favors the migration of erythrocytes into the space of Dissé and formation of perisinusoidal fibrosis. In this hypoxic situation, an increase of angiogenic factors (and activation of metalloproteinases in turn increasing vascular damage is induced. Second, nodular regenerative hyperplasia is favored by the chronic hypoxia of the centrilobular areas. Third, oxaliplatin can generate an obliteration of the blood capillaries and areas of parenchymal extinction that interrupt portal circulation and eventually elevate portal pressures.

There are three mechanisms through which oxaliplatin can cause sinusoidal damage, see Figure 1.

(a) Oxaliplatin can potentially cause increased porosity of the sinusoidal endothelium and increased cellular fenestrations, stimulating the release of free radicals and depletion of glutathione transferase, followed by an increase of metalloproteinases (MMP-2 and MMP-9) that also degrades the extracellular matrix in the space of Disse, causing the endothelial lining to aggregate. The damage in the sinusoidal endothelium favors the passage of erythrocytes into the space of Dissé and formation of perisinusoidal fibrosis. This hypoxia situation, in turn, generates an increased release of angiogenic factors (vascular endothelial factor, plasminogen-activating factor) and activation of metalloproteinases in turn increasing vascular damage [19].

(b) The appearance of nodular regenerative hyperplasia is favored by the chronic hypoxia of the centrilobular areas [18].

(c) Finally, oxaliplatin can generate an obliteration of the blood capillaries and areas of parenchymal extinction [19]. In this proposed model, the sinusoidal endothelial cells then dislodge, obstruct the sinusoids and interrupt portal circulation. The result is hepatic congestion and eventually elevated portal pressures.

Oxaliplatin effects on mitochondria have been suggested as a potential pathogenic mechanism in oxaliplatin associated neuropathy. Oxaliplatin creates mDNA adducts, which disturb mitochondrial protein synthesis and generate mitochondrial disfunction [20]. The exposure to high oxaliplatin concentrations increases the mitochondrial reactive oxygen species levels in neuronal cultures, proving that a mitochondrial oxidative stress may play an important role in the pathogenesis of oxaliplatin-induced peripheral neuropathy. This fact has not been studied in pathogenesis of porto sinusoidal vascular disease associated to oxaliplatin [21].

These three facts, can cause in a minority of cases the appearance of a presinusoidal increase of portal pressure (PP); this typically appears years after the completion of chemotherapy and manifests clinically as a typical portal hypertension (varices, ascites, splenomegaly and thrombocytopenia) [22].

The literature that has explored this fact is very scarce, with series of isolated cases and little homogeneity in the diagnosis of PSVD, sometimes confused with SOS. Risk factors are unknown [23,24]. However, an interesting study, published in 2010, related the relationship between the dose of oxaliplatin with the increase spleen size and thrombocytopenia during treatment with the development of SOS. In this cohort of patients, an increase of 50% or more of the spleen at the end of chemotherapy is associated with moderate to severe sinusoidal damage in 55% of patients undergoing hepatic metastasectomy [25]. In addition, although thrombocytopenia during treatment is very common, and secondary to medullar suppression and immune mechanisms, it typically reverts at the end of the treatment, while the persistence of a low platelet count should raise the suspicion of damage sinusoidal [26]. Between potencial risk factors associated to PSVD associated to oxaliplatin, are age, female sex, neurotoxicity, non resecable metastasis, preIQ elevated liver tests and metacronic or sincronic tumor [23]. Additionally, it has been suggested that antiangiogenic factors such as bevacizumab could protect from the development of the liver injury, because it blocks vascular endothelial growth factor (VEGF) [27,28,29]. Studies investigating liver samples from patients who have developed oxaliplatin-induced sinusoidal injury and a preclinical mouse model of sinusoidal injury using monocrotaline have demonstrated upregulation of VEGF-A as a key component of this toxicity [28]; thus, using VEGF tyrosine kinase inhibitors has demonstrated to have a protective effect with regards to monocrotaline-induced sinusoidal obstructive syndrome [25]. The protencial protective effect of bevacizumab has been suggested in a recent cohort of more than 200 patients [29].

### 2.2. When Should It Be Suspected?

PSVD associated or not to oxaliplatin (PSVD-OX), could be suspected in three different stages with different clinical manifestations depending on presence of portal hypertension. We can describe three different stages as follows: (1) patients without clinical signs of portal hypertension and histological findings that fits with PSVD, (2) asymptomatic patients with clear signs of portal hypertension and (3) patients with complications related to portal hypertension (such as ascites and variceal bleeding).

In the scarce literature, at time of diagnosis of IPH related to oxaliplatin (PSVD-OX), the majority of patients have been presented signs of portal hypertension for years, with splenomegaly and thrombocytopenia being the most frequent signs. Variceal bleeding is the most frequent initial clinical manifestation. Hemorrhage by peristomal varices is not uncommon in this group of patients [1,2,7]. Usually, patients have preserved liver function at the time of diagnosis; the appearance of ascites, although rare, is a factor of poor prognosis [30]; moreover, hepatic encephalopathy is very infrequent and is usually due to the presence of large portosystemic collaterals. Portal vein thrombosis is a frequent complication occurring in 13–45% of patients during follow-up [7]. Mortality from complications of portal hypertension, mainly variceal bleeding, is significantly lower than in cirrhotic patients, because of the preserved liver function. Mortality was commonly associated with concomitant comorbidities and not related to liver decompensation.

PSVD, associated with oxaliplatin, must be suspected in patients treated with this chemotherapy and clinical of radiological findings of portal hypertension, and non-hepatologist clinicians must be advised that not everything is cirrhosis.

### 2.3. How Can We Diagnose It?

Currently, there is no gold standard for diagnosis of IPH; diagnosis is based on the exclusion of other liver diseases. As commented above, in the vast majority of cases, the disease was diagnosed when clinical manifestations of portal hypertension were present.

A recent expert meeting of the VALDIG Consortium (Vascular Liver Disease Group) proposed the following criteria to support IPH diagnosis. They are summarized in Table 1 [5,30] and are based on noninvasive and invasive tests.

#### 2.3.1. Non Invasive Diagnosis

##### Laboratory

Anemia, thrombocytopenia and leukopenia, as markers of hypersplenism, are present in up to 87% of patients. The severity of these findings depends on the degree of portal hypertension [30]. Liver function tests are mostly normal, and liver function is preserved.

Only two studies were evaluated using a metabolomic profile with potential diagnostic or prognostic values. The small sample size of these studies, and the fact than only patients with portal hypertension were included, does not allow its application as an early diagnosis of the disease.

The number of patients in these studies was relatively small and all included patients had portal hypertension, thus, more studies are needed especially in patients without portal hypertension [31].

##### Imagin

Radiological features of PSVD include signs of portal hypertension (splenomegaly and porto-systemic collaterals). However, these features could be missing in patients in early stages of the disease (without clinical significant portal hypertension). The liver surface could be nodular and an hypertrophy of segment IV and I is found in patients with IPH [32,33]. However, these features are not fully specific. Splenomegaly is constant.

The absence of thrombosis in hepatic and portal veins must be as a prerequisite for the diagnosis of porto-sinusoidal vascular disease. The incidence of portal thrombosis during the evolution of the disease is high, therefore, in cases where the debut of the disease is associated with thrombosis of the portal splenic axis, since we cannot rule out that it is a purely prehepatic portal hypertension, they should be ruled out as a diagnosis of EVPS.

##### Elastography

In non-cirrhotic patients, mean liver stiffness is significantly lower than in cirrhotic patients with portal hypertension, and higher than in non- cirrhotic portal vein thrombosis (8.4 kPa ± 3.3 versus 40.9 kPa ± 20.5] versus 6.4 kPa ± 2.2) [33].

#### 2.3.2. Invasive Diagnosis

##### Liver Biopsy

**Figure 2:**
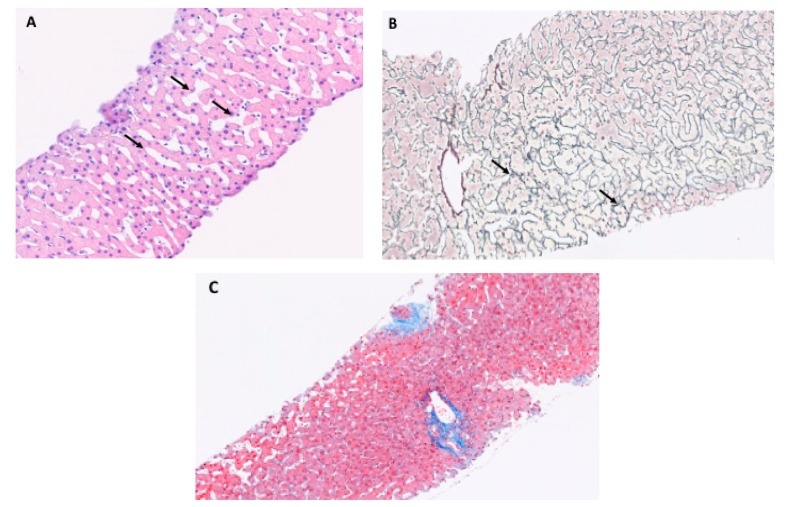
Histopathology of porto-sinusoidal vascular disease. (**A**) Section of liver biopsy at 10× magnificiation with hematein eosin with diffuse sinusoidal dilatation (arrows), (**B**) section of liver biopsy at 10× magnification with argentic reticulin stain with non-fibrous parenchyma preferentially around portal tracts (arrows), (**C**) Normal liver without signs of porto sinusoidal vascular disease.

There are three tipical histological alterations associated with PSVD: obliterative portal venopathy, nodular regenerative hyperplasia and incomplete septal cirrhosis [34]. They can be observed isolated or combined: (1) obliterative portal venopathy and hepatoportal sclerosis that affect small and medium branches of the portal vein. Phlebosclerosis is the most important feature, characterized by deposition of connective tissue around the vessels with irregular wall thickening and eccentric narrowing of vessel lumen, until the vein is occluded and collapsed; (2) nodular regenerative hyperplasia is based on the transformation of liver tissue into undefined small nodules (1–3 mm ); (3) these rudimentary nodules are surrounded by incomplete septal fibrosis (the third key feature) that are typically incomplete, thin, perforated or blind-ended septa [35,36], see Figure 2.

##### Hepatic Vein Catheterization

Patients with IPH typically present a HVPG below that of patients with cirrhosis; however, this finding is not totally specific, which is why recently, it has been proposed that HVPG measurements are not routinely needed in the evaluation of patients suspected to have IPH [37]. However, hepatic vein catheterization reveals important information, such as the presence of hepatic vein-to-vein communications, in more than 50% of patients with IPH (significantly higher than in patients with cirrhosis less than 10%); this supports the clinical suspicion of IPH [38].

##### Endoscopic Findings

Esophageal varices are present in about 80% to 90% of patients and compared with cirrhotic patients are larger. (90% versus 70%) and gastroesophageal varices (GOV 1 and GOV2) (31–44% versus 22%) and ectopic varices (included stomal varices) (89% versus 56%) are more prevalent. Portal hypertensive gastropathy is less common (5.4% versus 10.9%). Again, these findings are not found in early stages of the disease [39].

### 2.4. Management of the Disease

The management of complications of portal hypertension is under the same recommendations and guidelines that in patients with cirrhosis. [40]. Varices must be treated with are beta-blockers (propranolol and carvedilol) and endoscopic band ligation along or in combination depending on primary or secondary prophylaxis scenario [41]. Transjugular intrahepatic portosystemic shunts can be placed in patients with severe complications of portal hypertension [42]. For treatment of ectopic varices, including stomal varices embolization can be considered [43]. Likewise, splenectomy has been proposed for patients with severe hypersplenism [5].

Routinely anticoagulation is not recommended, except in cases of portal thrombosis or high-risk prothrombotic disorders [44]. Data on liver transplant in patients with IPH are scare, but favorable survival has been reported [45]. However, we have to be aware of the oncological comorbidities that are present in these patients and the outcome of them.

## 3. Conclusions

The lack of conclusive data regarding IPH-OX and the low incidence of the disease requires the use of extensive and rigorous records, which allows us to establish and clarify the natural history of the disease and try to identify clinical, analytical or radiological sings during the treatment or the follow-up to identify those patients at risk of developing the disease.

## Figures and Tables

**Table 1:** VALDIG criteria for the diagnosis of portal sinsusoidal vascular disease.

**Clinical Signs of Portal Hypertension (Any One of the Following †)** • Splenomegaly or hypersplenism • Esophageal varices • Ascites (non-malignant) • Minimally increased hepatic venous pressure gradient • Portovenous collaterals
**Exclusion of Cirrhosis on Liver Biopsy**
Exclusion of chronic liver disease causing cirrhosis or non-cirrhotic portal hypertension ‡ • Chronic viral hepatitis B or C • Non-alcoholic or alcoholic steatohepatitis • Autoimmune hepatitis • Hereditary hemochromatosis • Wilson’s disease • Primary biliary cholangitis
**Exclusion of Conditions Causing Non-Cirrhotic Portal Hypertension** • Congenital liver fibrosis • Sarcoidosis • Schistosomiasis
**Patent Portal and Hepatic Veins (Doppler Ultrasound or CT Scanning)**

All criteria must be fulfilled to diagnose portal sinusoidal vascular disease † Splenomegaly must be accompanied by additional signs of portal hypertension to fulfil this criterion. ‡ Chronic liver disease must be excluded, because severe fibrosis might be understaged on liver biopsy Addapted from De Gottardi et al. [5].

## Abbreviations

sinusoidal obstruction syndrome (SOS), portal pressure (PP), portal pressure gradient (HVPG), liver stiffness measurement (LSM), oxaliplatin (OX), idiophatic portal hypertension (IPH), portal hypertension (PHT).
